# Randomized Controlled Trial of Mindfulness-Based Stress Reduction Versus Aerobic Exercise: Effects on the Self-Referential Brain Network in Social Anxiety Disorder

**DOI:** 10.3389/fnhum.2012.00295

**Published:** 2012-11-05

**Authors:** Philippe Goldin, Michal Ziv, Hooria Jazaieri, James J. Gross

**Affiliations:** ^1^Department of Psychology, Stanford UniversityStanford, CA, USA

**Keywords:** social anxiety, self-view, mindfulness, fMRI, exercise, brain, self, meditation

## Abstract

**Background:** Social anxiety disorder (SAD) is characterized by distorted self-views. The goal of this study was to examine whether mindfulness-based stress reduction (MBSR) alters behavioral and brain measures of negative and positive self-views. **Methods:** Fifty-six adult patients with generalized SAD were randomly assigned to MBSR or a comparison aerobic exercise (AE) program. A self-referential encoding task was administered at baseline and post-intervention to examine changes in behavioral and neural responses in the self-referential brain network during functional magnetic resonance imaging. Patients were cued to decide whether positive and negative social trait adjectives were self-descriptive or in upper case font. **Results:** Behaviorally, compared to AE, MBSR produced greater decreases in negative self-views, and equivalent increases in positive self-views. Neurally, during *negative* self versus case, compared to AE, MBSR led to increased brain responses in the posterior cingulate cortex (PCC). There were no differential changes for *positive* self versus case. Secondary analyses showed that changes in endorsement of *negative* and *positive* self-views were associated with decreased social anxiety symptom severity for MBSR, but not AE. Additionally, MBSR-related increases in dorsomedial prefrontal cortex (DMPFC) activity during *negative* self-view versus case were associated with decreased social anxiety related disability and increased mindfulness. Analysis of neural temporal dynamics revealed MBSR-related changes in the timing of neural responses in the DMPFC and PCC for *negative* self-view versus case. **Conclusion:** These findings suggest that MBSR attenuates maladaptive habitual self-views by facilitating automatic (i.e., uninstructed) recruitment of cognitive and attention regulation neural networks. This highlights potentially important links between self-referential and cognitive-attention regulation systems and suggests that MBSR may enhance more adaptive social self-referential processes in patients with SAD.

## Introduction

Self-views can powerfully influence how a person thinks, feels, and behaves, particularly in social contexts. The relationship of self-views to social functioning is especially salient in the clinical context of social anxiety disorder (SAD). Cognitive models of social anxiety (Clark and Wells, 1995) suggest that distorted self-views, specifically, regarding the self as socially awkward, inadequate, or flawed are an essential feature of SAD. Recent conceptualizations highlight distorted social self-views as the core problem in SAD (Moscovitch, 2009). These models suggest that maladaptive social self-reflective processes are implicated in cognitively biased evaluations of self which generate negative emotions, disrupt emotion regulation, and interfere with social self-efficacy and performance (Spurr and Stopa, 2002).

**Figure 1:**
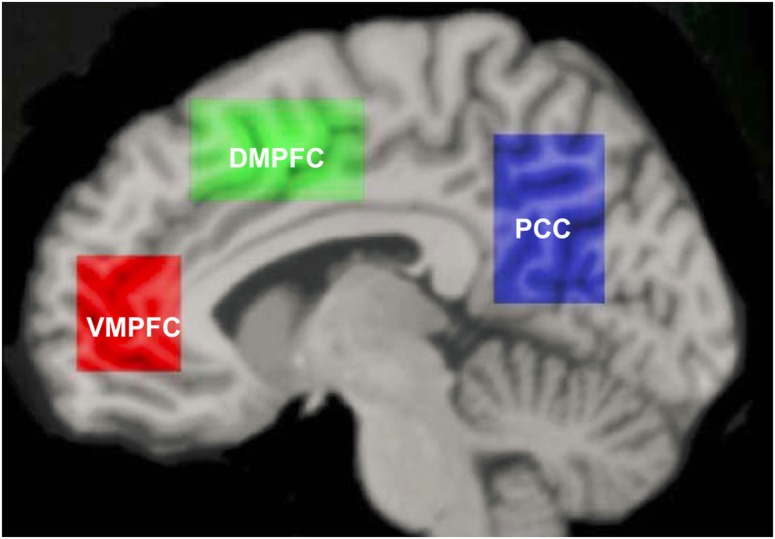
**Self-referential network from Northoff et al. (2006)**.

Meta-analyses of neuroimaging studies of self-referential processing in healthy adults have identified a set of three cortical midline brain regions that comprise the self-referential network (SRN), including the ventromedial prefrontal cortex (VMPFC), dorsomedial prefrontal cortex (DMPFC), and posterior cingulate cortex/precuneus (hereafter abbreviated as PCC; Figure 1; Northoff et al., 2006, 2011). To induce activation in the SRN, studies have used judgments of visually presented trait adjectives (Kelley et al., 2002), aurally presented statements (Johnson et al., 2002), or mental reflection on self-traits (Kjaer et al., 2002). Most studies have used tasks that require making a rapid judgment of whether or not a specific trait applies to oneself. Specifically, neural activity in the VMPFC has been shown to be related to ratings of self-relatedness (Phan et al., 2004; de Greck et al., 2008). Activation of the DMPFC and PCC has been observed during self-endorsement of positive and negative traits in healthy adults (Fossati et al., 2003).

Neuroimaging studies of self-referential processing have begun to investigate abnormal patterns of SRN activity in a variety of clinical samples, however, results are mixed. In patients with major depressive disorder, fMRI investigations have reported elevated VMPFC activation during event-related task designs which is thought to be associated with more automatic alerting to self-relevant information, as well as elevated transient DMPFC activation during block task designs which may represent more strategic evaluation (Lemogne et al., 2012). In patients with SAD, fMRI investigations have observed SRN activation during self-referential processing of trait adjectives, as well as brain regions implicated in language, memory, affective, and visual processing during social trait self-endorsement (Goldin et al., 2009a), and decreased VMPFC response for first (versus second) person self-referential statements which were associated with increased social anxiety symptom severity (Blair et al., 2011). However, there are no randomized controlled trials (RCT) that have compared the impact of different psychosocial interventions on SRN activity in patients with SAD. Conducting such RCTs may provide stronger evidence for the function and modulation of the SRN in clinical samples.

One especially intriguing psychosocial intervention that directly cultivates non-identification with self-views is mindfulness-based stress reduction (MBSR; Kabat-Zinn, 1990). Meta-analyses of MBSR training in healthy adults have shown reliable reductions in symptoms of anxiety, stress, and ruminative thinking (Chiesa and Serretti, 2009). Among patients with anxiety disorders, meta-analysis indicates robust reduction of anxiety, and depression symptoms (Vøllestad et al., 2012). An important fMRI study of self-reference in a non-clinical sample of adults found that for the contrast of experiential (versus narrative) self-focus MBSR resulted in brain response decreases in anterior VMPFC and DMPFC regions and increases in a right lateralized network including dorsolateral and ventrolateral PFC, insular cortex, supramarginal gyrus, angular gyrus, inferior parietal lobule suggesting suppression of conceptual narrative self-processing, and enhancement of viscerosomatic processing and present-moment focused attention (Farb et al., 2007). Inferences from this study are limited because brain responses were measured only at post-MBSR. In the few studies of patients with SAD, there are preliminary positive results for MBSR training, including reduction of clinical symptoms and increased well-being (Koszycki et al., 2007; Jazaieri et al., 2012). Proposed mechanisms of MBSR include modulation of attention regulation, body awareness, emotion regulation, and self-views (Hölzel et al., 2011). Thus, MBSR is expected to decrease grasping at conceptual self-views (instantiated in the SRN and language brain networks) and to increase present-moment experiential modes of self-processing (instantiated in viscerosomatic networks). The impact of MBSR on the SRN in clinical populations, however, is not yet understood.

In patients with SAD, MBSR has been shown to modulate attention regulation, emotion regulation, and self-views (Goldin et al., 2009a, 2012; Goldin and Gross, 2010). Neurally, during self-referential processing in patients with SAD, MBSR has been shown to *decrease* neural responses in brain regions related to self-referential and language processing for *positive* self-views, and *increase* responses in brain regions linked to attention engagement and regulation for *negative* self-views (Goldin et al., 2009a). This study also reported associations of pre-to-post-MBSR increases in attention engagement related parietal brain regions and decreases in anxiety symptoms. However, important caveats of these studies include a small sample size, participant self-selection, and no active comparison intervention. Thus, the implementation of a RCT with two active and dose matched stress reduction programs should support stronger inferences about the effects of MBSR on self-views.

In addition to modulation of brain response magnitude, there is growing evidence of abnormal neural temporal dynamics during social cognition and emotion regulation in patients with SAD (Campbell et al., 2007; Goldin et al., 2009b). One study has reported MBSR-related changes in neural temporal dynamics of emotional reactivity to negative self-views in adults with SAD (Goldin and Gross, 2010). Currently, nothing is known about how MBSR impacts the neural temporal dynamics of brain responses within the SRN. Analysis of neural temporal dynamics might elucidate the effects of MBSR on other cognitive processes that come online in patients with SAD when engaging to self-referential processing, such as shifts in attention regulation and cognitive regulation of emotional reactivity to self-views.

Considered together, these changes suggest that MBSR likely modulates multiple cognitive processes. MBSR is hypothesized to increase the ability to decenter from habitual patterns of reactivity. However, in the case of adults with SAD, MBSR may have more specific effects such as modifying some of the core cognitive biases (e.g., distorted self-views, maladaptive attention, and emotional processing, deficient cognitive regulation) that are fundamental features of social anxiety.

The goal of the present study was to investigate the differential effects of MBSR versus an active control condition [aerobic exercise (AE) stress reduction] on the neural bases of self-referential processing in patients with generalized SAD in the context of a RCT. MBSR cultivates a non-judgmental, pliable present-moment awareness that decreases habitual patterns of evaluation and reactivity, and increases psychological flexibility. In patients with SAD, MBSR is associated with reduced symptoms of anxiety, depression and self-deprecation, and increased quality of life and functionality (Koszycki et al., 2007). A recent review suggests that the brain-based mechanisms underlying MBSR include attention regulation, body awareness, emotion regulation, and changes in the self-views (Hölzel et al., 2011). Few studies, however, have investigated these proposed brain-based mechanisms in the context of a RCT. To do this, we choose AE as a control condition for several reasons. Like MBSR, AE has been shown to improve both physical and mental health (Penedo and Dahn, 2005), especially symptoms of stress, depression, and anxiety (Petruzzello et al., 1991; Stich, 1998; Ströhle, 2009) in clinical samples of mixed anxiety (e.g., Merom et al., 2007), panic disorder (e.g., Broocks et al., 1998; Dratcu, 2001), and generalized anxiety disorder (e.g., Steptoe et al., 1989; McEntee and Halgin, 1999). As implemented in this study, we matched the amount of individual and group practice for MBSR and AE. Presently, there are no studies describing the impact of AE on self-views in patients with SAD.

We conducted a RCT in which we obtained behavioral and neural measures of self-views during an fMRI assessment in patients with generalized SAD before and after MBSR or AE. For behavioral responses, we expected that, compared to AE, MBSR would result in self-endorsement of more adaptive self-views, namely, greater *decreases* in negative and greater *increases* in positive self-views. For neural responses, based on prior studies that reported reduction in cortical midline brain region responses during experiential (versus narrative) self-focus (Farb et al., 2007) and during self-referential processing in patients with SAD (Goldin et al., 2009a), we expected that, compared to AE, MBSR would result in greater *reductions* during negative and positive self-referential processing in the VMPFC and DMPFC. Based on previous reports of MBSR-related increased activity in parietal cortex regions associated with attention engagement and regulation in general (Farb et al., 2007; Hölzel et al., 2011) and specifically in patients with SAD (Goldin et al., 2009a; Goldin and Gross, 2010), we expected that, compared to AE, MBSR would result in greater *increases* in PCC during negative and positive self-referential processing. In secondary analyses, we examined whether MBSR and AE-related changes in behavioral and brain responses related to changes in clinical symptoms and mindfulness, and how MBSR impacted the neural temporal dynamics in the three SRN brain regions.

## Materials and Methods

### Participants

**Figure 2:**
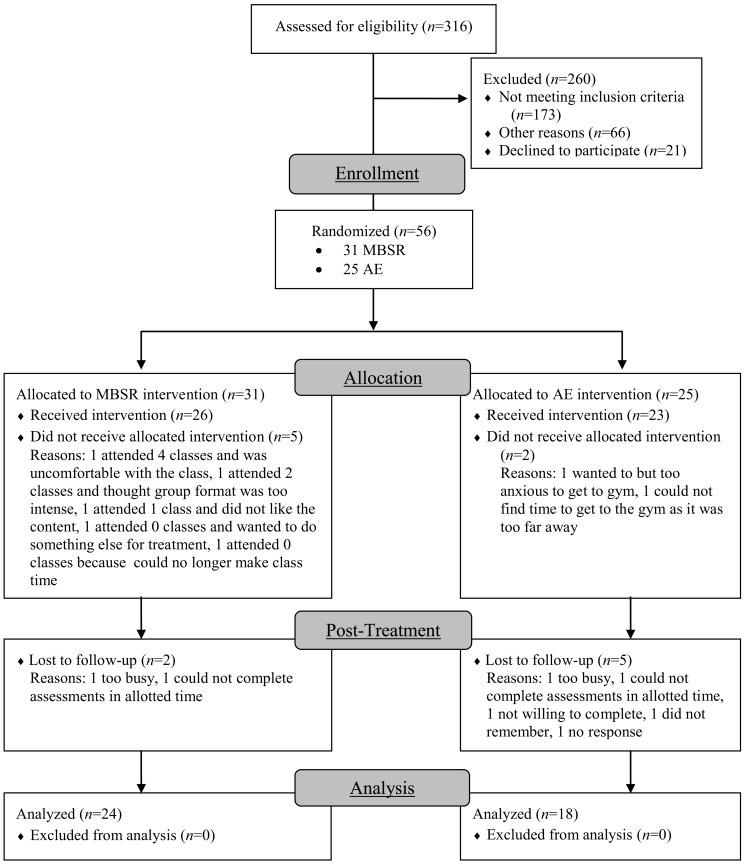
**Consolidated standards of reporting trials diagram for randomized controlled trial of mindfulness-based stress reduction (MBSR) versus aerobic exercise (AE)**.

Participants were unmedicated patients seeking treatment for SAD who met DSM-IV (American Psychiatric Association, 1994) criteria for generalized SAD. Of 316 individuals assessed for eligibility, 260 were excluded (173 for not meeting study criteria, 66 for other reasons, and 21 declined to participate). The remaining 56 participants were randomly assigned to either MBSR (*n* = 31) or AE (*n* = 25; Figure 2). Groups did not differ in gender, age, ethnicity, or education (Table 1). fMRI assessments at both baseline and post-intervention were available for 24 MBSR and 18 AE participants.

**Table 1:** **Demographic characteristics at baseline for patients randomized to mindfulness-based stress reduction versus aerobic exercise**.

	MBSR	AE	*t*-Test
	*n* = 31	*n* = 25	or χ^2^
Females, *n* (%)	19 (61.3%)	10 (40%)	χ^2^ = 2.88
Age (*M* years ± SD)	32.87 ± 8.83	32.88 ± 7.97	*t* = 0.42
Ethnicity, *n* (%)			χ^2^ = 0.43
Caucasian	13 (41.9%)	10 (40%)	
Asian	14 (45.2%)	11 (44%)	
Hispanic	3 (9.7%)	1 (4%)	
Multiracial	1 (3.2%)	3 (12%)	
Education (*M* years ± SD)	16.40 ± 2.00	16.84 ± 2.64	*t* = 0.34
Current axis-I comorbidity			χ^2^ = 4.88
Generalized anxiety disorder	10 (32%)	8 (32%)	
Major depressive disorder	5 (16%)	6 (24%)	
Dysthymia	2 (6%)	3 (12%)	
Specific phobia	3 (10%)	2 (8%)	
Panic disorder	2 (6%)	2 (8%)	
Agoraphobia	1 (3%)	2 (8%)	
Obsessive-compulsive disorder	1 (3%)	0	
Past axis-I comorbidity			χ^2^ = 1.49
Major depressive disorder	9 (29%)	2 (8%)	
Dysthymia	1 (3%)	0	
Panic disorder	1 (3%)	0	
Obsessive-compulsive disorder	0	1 (4%)	
Substance abuse	0	1 (4%)	
Eating disorder	3 (10%)	1 (4%)	
Past psychotherapy	15 (48%)	9 (36%)	χ^2^ = 1.84
Past pharmacotherapy	7 (23%)	5 (20%)	χ^2^ = 1.56
GAF (*M* ± SD)	53.36 ± 5.87	55.38 ± 4.06	*t* = 1.02
CGI (*M* ± SD)	4.93 ± 1.23	5.22 ± 0.60	*t* = 1.33

**n*, Sample size, *M*, mean; SD, standard deviation; GAF, global assessment of functioning; CGI, clinical global impression*.

Patients passed an MR safety screen and had to be medication and psychotherapy-free for at least 6 months with no history of medical disorders or head trauma. Patients had to have a primary diagnosis of generalized SAD and no evidence of thought disorders, bipolar depression, alcohol, or drug dependence. Patients were not permitted to be currently in any form of pharmacological or psychological treatment. Patients were excluded if they had previously completed an MBSR course or if they had a regular meditation practice or AE regime (defined as three or more times per week, for more than 2 months). Patients randomized to MBSR and AE did not differ in current or past Axis-I comorbidity, current Global Assessment of Functioning or Clinical Global Impression, and past psychotherapy or past pharmacotherapy (Table 1).

### Procedure

Patients were recruited through web-based community listings and referrals from mental health providers. After passing a telephone screening, potential participants were administered the ADIS-IV-L (Di Nardo et al., 1994) by a clinical psychologist. We enrolled patients with a principal diagnosis of generalized SAD operationalized as (a) moderate or higher severity (≥4 on a scale of 0–8) on the ADIS-IV-L Clinician’s Severity Rating for SAD and (b) social fear for five or more distinct social or performance situations in the SAD section. Patients were randomly assigned to MBSR or AE using a biased coin randomization procedure (Efron, 1971) which entails higher probability of allocation to the group with fewer participants. This method controls for potential confounds encountered when a greater number of participants are assigned at specific time points during a trial to one of two interventions. Patients were administered self-report clinical and fMRI scanning assessments before and after MBSR and AE. All participants provided informed consent in accordance with the Stanford University Human Subjects Committee and federal guidelines.

### Mindfulness-based stress reduction

Mindfulness-based stress reduction consisted of eight, weekly 2.5 h group classes, a 1-day meditation retreat, and daily home practice. Patients were trained in formal meditation, informal practice, and Hatha yoga. Daily logs were collected each week to measure group and individual meditation and yoga practice. Patients attended MBSR courses offered by seven different teachers in eight healthcare settings throughout the San Francisco Bay Area. Instructors had an average of 15.7 years (SD = 4.1 years, range = 10–20 years) of MBSR teaching experience.

### Aerobic exercise

To match the individual and group components of MSBR, participants in the 8-week AE intervention were provided a 2-month gym membership and were required to complete weekly at least two individual AE sessions and one group AE session (other than meditation or yoga).

### Self-referential encoding task

The self-referential encoding task (SRET; Derry and Kuiper, 1981) is considered an information processing measure of self-schema. Stimuli consisted of 25 positive and 25 negative social trait adjectives from the Affective Norms of Emotion Words database (Bradley and Lang, 1999), balanced (all *p*s > 0.51) on word frequency (positive adjectives = 40.5, negative adjectives = 33.6) and number of letters (positive adjectives = 6.9, negative adjectives = 7.2), as well as on arousal (positive adjectives = 5.54, negative adjectives = 5.43 on a scale of 1 = low to 9 = high using) and valence (deviation from neutral: positive adjectives = 2.66, negative adjectives = 2.58 on a scale of 1 = most negative, 5 = neutral, 9 = most positive) based on the nine-point Self-Assessment Manikin rating system (Lang, 1980).

**Figure 3:**
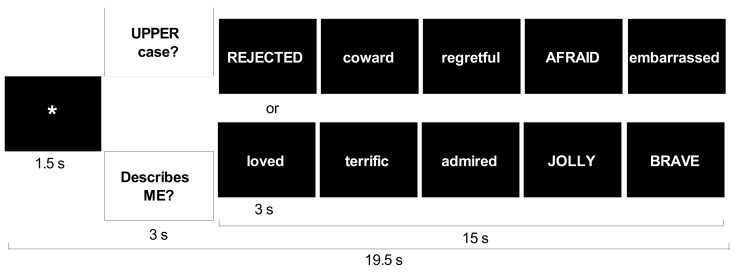
**Trial structure for self-referential encoding task**.

The SRET was 5 min and 39 s in length. Each adjective was presented twice, once in each of two conditions. The self-referential condition assessed self-focused social-evaluative processing. Case identification was used as a comparison condition to control for reading negative and positive adjectives while determining whether the word consisted of upper or lower case letters. Each of the four trial types (two conditions by two valences) included five blocks. Each block consisted of fixation, question (either “Describes ME?” or “UPPER case?”), and five adjectives of the same valence presented one at a time for 3 s each (Figure 3).

Stimulus order included a pseudo-random block sequence with no more than two blocks of the same condition in a row and a random sequence of words and upper/lower case within each block. Patients pressed buttons to indicate whether or not a word was self-descriptive or in uppercase letters.

### Measures of clinical symptoms and mindfulness

To measure severity of social anxiety symptoms, we used the 24-item *Liebowitz Social Anxiety Scale-Self-Report* (LSAS-SR; Liebowitz, 1987; Fresco et al., 2001), which consists of questions that assess social interaction situations (11 items) and performance situations (13 items). A four-point Likert-type scale is used for ratings of fear and of avoidance, with a range from 0 (*none* and *never*, respectively) to 3 (*severe* and *usually*, respectively) for situations during the past week. Ratings are summed for a total LSAS-SR score (range = 0–144). The LSAS-SR has reliability and construct validity (Rytwinski et al., 2009) and its internal consistency (Cronbach’s alpha) for this study was 0.90 in MBSR and 0.90 in AE.

To measure social anxiety related disability, we administered the *Sheehan Disability Scale* (SDS; Sheehan, 1983) which measures work, social life, and family life impairment as a function of SAD and comorbid conditions, and has demonstrated good internal consistency and validity (Hambrick et al., 2004), and its internal consistency in this study was 0.68 in MBSR and 0.68 in AE.

To measure mindfulness, we used the *Kentucky Inventory of Mindfulness Skills* (FFMQ; Baer, 2003). It is a 39-item self-report measure which measures mindfulness, and includes four components: observing, describing, acting with awareness, and accepting without judgment. Items are rated on a five-point Likert scale. The instrument has good internal consistency and validity (Baer et al., 2004), and its internal consistency in this study was 0.76 in MBSR and 0.85 in AE.

To measure the potential confound of social desirability, we administered the 10-item *Marlowe–Crowne Social Desirability Scale* (MCSDS; Crowne and Marlowe, 1960). The instrument consists of true-false items with four reverse coded items, with higher scores reflecting a greater tendency to give a socially desirable response. It has shown adequate internal consistency and reliability (Crino et al., 1983).

### fMRI acquisition

Imaging was performed on a General Electric 3 T Signa magnet using a custom-built quadrature “dome” elliptical bird cage head coil and a T2*-weighted gradient echo spiral-in/out pulse sequence that used blood oxygenation level-dependent (BOLD) contrast. Head movement was minimized using a bite bar and padding. During a single run, 226 volumes (each consisting of 22 sequential axial slices) were obtained (TR = 1500 ms, TE = 30 ms, flip angle = 60, FOV = 22 cm, frequency encoding = 64, voxel = 3.438 mm × 4.5 mm). A high-resolution anatomical scan was acquired using a fast spin-echo spoiled grass pulse sequence (voxel = 0.85942 mm × 1.2 mm; FOV = 22 cm, frequency encoding = 256).

### fMRI data preprocessing

Analysis of functional neuroimages (AFNI) software (Cox, 1996) was used for preprocessing and statistical analysis. Preprocessing included an analysis of potential outliers, volume registration to a base image, motion correction, 4 mm^3^ isotropic Gaussian spatial smoothing, high-pass filtering (0.011 Hz), linear detrending, and conversion into percentage change in each voxel. No volumes demonstrated motion in the *x*, *y*, or *z* directions in excess of ±0.8 mm. There was no stimulus-correlated motion, as assessed by correlations between condition-specific reference functions and *x*, *y*, *z* motion correction parameters (all *p*s > 0.48).

### fMRI statistical analysis

Multiple-regression was implemented with AFNI 3dDeconvolve and included baseline parameters to remove mean, linear, and quadratic trends, and motion-related variance in the BOLD signal. Regressors for the negative self, negative case, positive self, and positive case were convolved with the gamma variate model (Cohen, 1997) of the hemodynamic response function. Functional MRI BOLD signal intensity was computed as percentage of signal change [(MR signal per voxel per time point/mean MR signal in that voxel for the entire functional run) × 100]. Brain maps were converted to Talairach atlas space (Talairach and Tournoux, 1988) and second-level group statistical parametric maps were produced according to a random-effects model.

Repeated-measures analysis of variance with planned follow-up *t*-tests was conducted to examine average BOLD responses in masks for each of the three cortical midline SRN regions (VMPFC, DMPFC, and PCC). The *a priori* rectangular region-of-interest masks were defined by a meta-analysis of 27 neuroimaging studies (Northoff et al., 2006). The Talairach coordinates of the boundaries of the three rectangular masks are: VMPFC *x* = −12 to 8, *y* = 38–60, *z* = −7 to 21, DMPFC: *x* = −14 to 12, *y* = −11 to 31, *z* = 37–61, and PCC: *x* = −14 to 8, *y* = −74 to −48, *z* = 10–52.

For the examination of pre-to-post-intervention changes in the temporal dynamics in each region we used repeated-measures ANOVA with the Greenhouse–Geisser correction for potential violations of sphericity in the time course of 10 time points (or 15 s per trial).

We also conducted whole-brain *t*-tests to examine within-group changes pre-to-post-MBSR and AE, separately, on negative and positive self-views. We used AlphaSim, a Monte Carlo simulation bootstrapping program in the AFNI library, to protect against false positive cluster detection (Forman et al., 1995). The program determined that a voxel-wise *p* < 0.001 threshold and cluster volume threshold of ≤213 mm^3^ (4 voxels × 3.438 mm × 4.5 mm) provided protection against false positive cluster detection at *p* < 0.01.

## Results

### Clinical responses

As shown in Table 2, both MBSR and AE yielded significant reductions in social anxiety symptom severity (LSAS-SR; *t*s > 4.05, *p*s < 0.001), SAD-related disability (SDS; *t*s > 2.29, *p*s < 0.05), and increases in mindfulness (KIMS; *t*s > 2.26, *p*s < 0.05). However, there were no between-group differences in pre-to-post change (all *p*s > 0.34). A full account of the treatment-related self-reported changes in clinical and well-being measures has been published elsewhere (Jazaieri et al., 2012).

**Table 2:** **Self-endorsement and clinical variables**.

	MBSR *n* = 31	AE *n* = 25
	Mean ± SD	Mean ± SD
**LIEBOWITZ SOCIAL ANXIETY SCALE-SELF-REPORT**		
Pre	87.3 ± 20.6	90.1 ± 17.1
Post	56.7 ± 19.0	61.4 ± 28.6
**SHEEHAN DISABILITY SCALE**		
Pre	17.8 ± 7.1	18.0 ± 6.8
Post	9.2 ± 3.8	13.5 ± 8.5
**KENTUCKY INVENTORY OF MINDFULNESS SKILLS**		
Pre	110.9 ± 12.9	110.9 ± 14.9
Post	126.5 ± 13.2	117.5 ± 14.6
**NEGATIVE SELF-ENDORSEMENT**		
Pre	66.8 ± 18.4	65.3 ± 18.7
Post	33.6 ± 22.0	49.8 ± 27.0
**POSITIVE SELF-ENDORSEMENT**		
Pre	32.3 ± 16.7	35.5 ± 20.3
Post	50.7 ± 28.1	46.2 ± 20.0

To rule out the possibility of a social desirability response bias on self-report measures, we examined the relationship of the MCSDS and the clinical variables listed in Table 2. We found no relationship (*r*s: −0.20 to +0.11, all *p*s > 0.09) between the MCSDS and *baseline* SRET negative self-endorsement, SRET positive self-endorsement, social anxiety symptom severity (LSAS), SAD-related disability (SDS), and mindfulness skills (Kentucky Inventory of Mindfulness Skills). There was also no relationship between the MC and the same measures at post-intervention (*r*s: −0.08 to 0.21, all *p*s > 0.21).

### Behavioral responses

**Figure 4:**
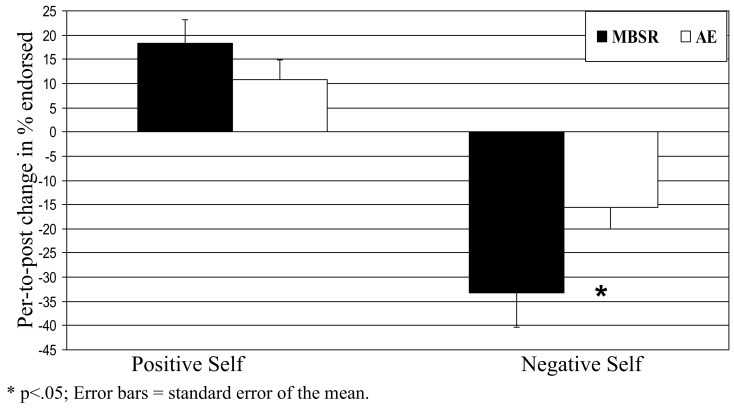
**Blood oxygenation level-dependent signal at baseline for negative self versus case and positive self versus case for all patients with SAD**. T-maps were thresholded at *t* > 3.65, voxel-wise *p* < 0.001 and cluster volume >6 voxels in order to obtain a cluster-wise *p* < 0.05. *p < 0.05; error bars = SEM.

For self-endorsement of *negative* social traits, a 2 Group (MBSR, AE) × 2 Time (pre, post) repeated-measures ANOVA yielded no main effect of group (*p* > 0.25), a main effect of time (*F* = 46.31, *p* < 0.0001, ${\text{η}}_{\text{p}}^{\text{2}}$ = 0.63), and an interaction of group by time (*F* = 4.50, *p* < 0.05, ${\text{η}}_{\text{p}}^{\text{2}}$ = 0.14). Follow-up paired *t*-tests showed *decreases* in negative self-endorsement from pre-to-post-MBSR (change = −33.2, *t* = 5.72, *p* < 0.001, ${\text{η}}_{\text{p}}^{\text{2}}$ = 0.70) and AE (change = −15.5, *t* = 3.84, *p* < 0.002, ${\text{η}}_{\text{p}}^{\text{2}}$ = 0.53). Compared to AE, MBSR led to greater decreases in negative self-endorsement (*t* = 2.10, *p* < 0.05, ${\text{η}}_{\text{p}}^{\text{2}}$ = 0.16; Figure 4; Table 2).

For self-endorsement of *positive* social traits, a 2 Group × 2 Time repeated-measures ANOVA yielded no main effect of group (*p* > 0.84), a main effect of time (*F*_1,28_ = 25.75, *p* < 0.001, ${\text{η}}_{\text{p}}^{\text{2}}$ = 0.49), and no interaction of group by time (*p* > 0.33). Follow-up paired *t*-tests revealed *increases* in positive self-endorsement from pre-to-post-MBSR (change = 18.4, *t* = 4.14, *p* < 0.001, ${\text{η}}_{\text{p}}^{\text{2}}$ = 0.51) and AE (change = 10.7, *t* = 3.02, *p* < 0.01, ${\text{η}}_{\text{p}}^{\text{2}}$ = 0.41) that were not statistically different (*t* = 0.97, *p* > 0.34).

### Brain responses

#### Manipulation check

**Figure 5:**
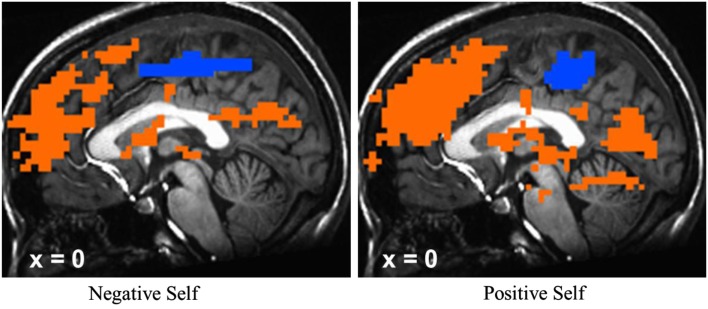
**Pre-to-post change in self-endorsement of negative and positive self-views**.

To confirm that the SRET task induced activation of the cortical midline SRN, we examined BOLD responses for negative and positive self-views, separately, across all patients at baseline. There was evidence of robust activations in VMPFC, DMPFC, and PCC regions for the contrasts of negative self versus case and positive self versus case (Figure 5).

#### Treatment-related changes in the self-referential network

We conducted a 2 Group (MBSR, AE) × 2 Time (pre, post) repeated-measures ANOVA of BOLD responses in each of the three SRN *a priori* ROIs (DMPFC, VMPFC, PCC) for the contrasts of *negative* self (versus case) and *positive* self (versus case), separately.

#### Negative self-view

**Figure 6:**
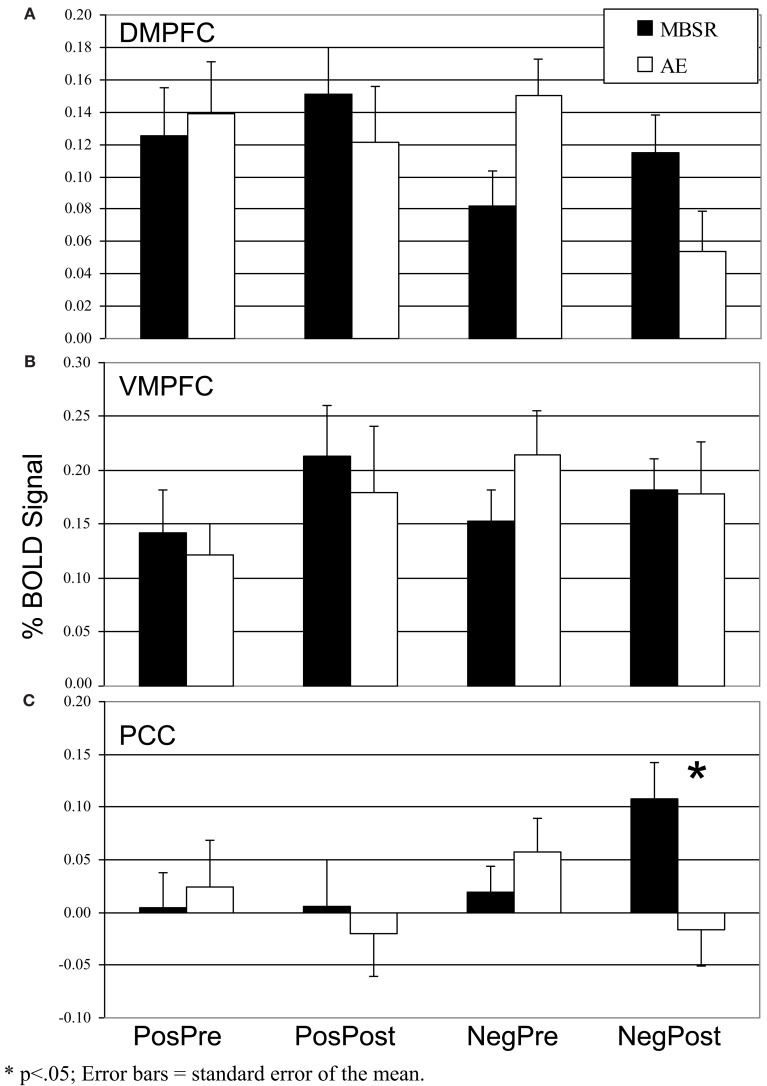
**Blood oxygenation level-dependent signal in the three self-referential network brain regions pre- and post-mindfulness-based stress reduction and aerobic exercise in patients with social anxiety disorder for positive self versus case and negative self versus case (A) Dorsomedial Prefrontal Cortex**. **(B)** Ventromedial Prefrontal Cortex. **(C)** Posterior Cingulate Cortex. *p < 0.05; error bars = SEM.

For the DMPFC, there were no main effects of time (*p* > 0.14) or group (*p* > 0.89), but there was a significant interaction of group by time (*F* = 8.87, *p* < 0.005, ${\text{η}}_{\text{p}}^{\text{2}}$ = 0.18). Follow-up paired *t*-tests showed no significant change in BOLD signal from pre-to-post-MBSR (change = 0.03, *p* > 0.29, ${\text{η}}_{\text{p}}^{\text{2}}$ = 0.4) and a decrease from pre-to-post-AE (change = −0.10, *t* = 3.62, *p* = 0.002, ${\text{η}}_{\text{p}}^{\text{2}}$ = 0.45; Figure 6). For the VMPFC, there were no main or interaction effects (all *p*s > 0.35). For the PCC, there were no main effects of time (*p* > 0.82) or group (*p* > 0.17), and a significant interaction of group by time (*F* = 6.29, *p* < 0.05, ${\text{η}}_{\text{p}}^{\text{2}}$ = 0.13). Follow-up *t*-tests showed an increase from pre-to-post-MBSR (change = 0.09, *t* = 2.16, *p* < 0.05, ${\text{η}}_{\text{p}}^{\text{2}}$ = 0.16) and no significant change in AE (change = −0.07, *p* > 0.15, ${\text{η}}_{\text{p}}^{\text{2}}$ = 0.12).

#### Positive self-view

There were no main or interaction effects (all *p*s > 0.10) in the VMPFC, DMPFC, and PCC.

### Secondary analyses

We examined whether MBSR and AE-related changes in behavioral and brain responses were related to changes in clinical symptoms and mindfulness, and also tested how MBSR impacted the neural temporal dynamics in the three SRN brain regions for the contrast of negative self versus case. For completeness, we also report the within-group effects of MBSR and AE, separately, on whole-brain responses for negative and positive self-referential processing.

#### Association of behavioral responses and clinical symptoms

For behavioral responses, decrease in self-endorsement of *negative* self-views was associated with decrease in social anxiety symptom severity (LSAS-SR) from pre-to-post-MBSR (*r* = 0.85, *p* < 0.001), but not AE (*r* = 0.00, *p* > 0.99; *Z*_diff_ = 2.48, *p* = 0.013). Increase in self-endorsement of *positive* self-views was associated with decrease in social anxiety symptom severity from pre-to-post-MBSR (*r* = −0.94, *p* < 0.001), but not from pre-to-post-AE (*r* = −0.11, *p* > 0.73; *Z*_diff_ = 3.25, *p* = 0.001). There was no relationship with mindfulness (KIMS).

#### Association of brain responses and clinical symptoms/mindfulness

**Figure A1:**
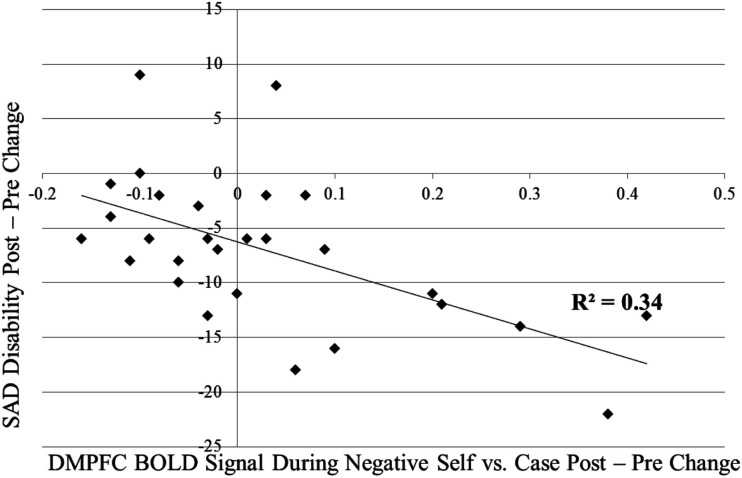
**Association of pre-to-post-MBSR changes in DMPFC during negative self versus case and social anxiety related disability**.

**Figure A2:**
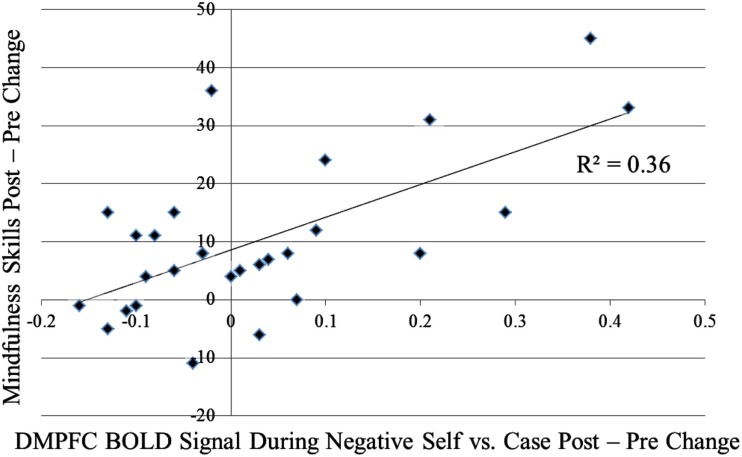
**Association of pre-to-post-MBSR changes in DMPFC during negative self versus case and mindfulness skills**.

**Figure A3:**
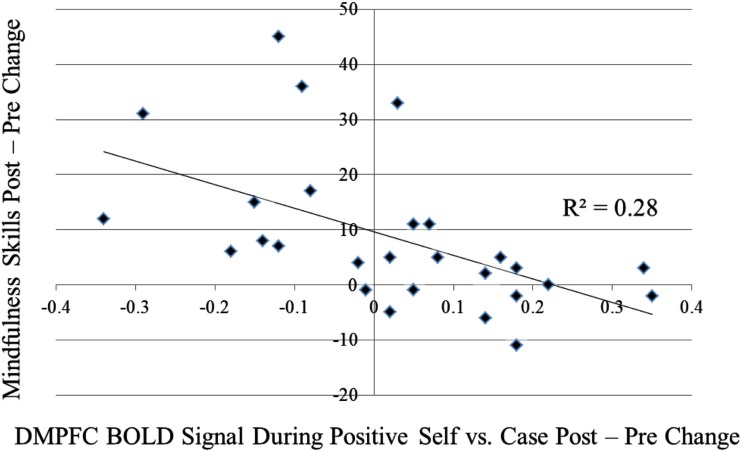
**Association of pre-to-post-MBSR changes in DMPFC during positive self versus case and mindfulness skills**.

For the contrast of self-endorsement of *negative* self (versus case), increases in DMPFC activity were associated with (a) decreased social anxiety related disability (SDS) from pre-to-post-MBSR (*r* = −0.58, *p* < 0.05; Figure A1 in Appendix) and AE (*r* = −0.63, *p* < 0.05) and (b) increased mindfulness (KIMS) in MBSR (*r* = 0.60, *p* < 0.05; Figure A2 in Appendix), but not in AE (*r* = 0.35, *p* > 0.43; *Z*_diff_ = 0.80, *p* > 0.42). For the contrast of self-endorsement of *positive* self (versus case), decreases in DMPFC activity were associated with increased mindfulness (KIMS) in MBSR (*r* = −0.53, *p* < 0.05; Figure A3 in Appendix) but not in AE (*r* = 0.21, *p* > 0.65; *Z*_diff_ = 1.98, *p* < 0.05).

#### Neural temporal dynamics

For the contrast of *negative* self (versus case) in the MBSR group, we conducted a 2 Group (MBSR, AE) × 2 Time (pre, post) × 10 TRs (ten 1.5 s TRs or brain volume acquisitions) repeated-measures ANOVA with Greenhouse–Geisser correction on BOLD responses for each of the three SRN brain regions.

**Figure 7:**
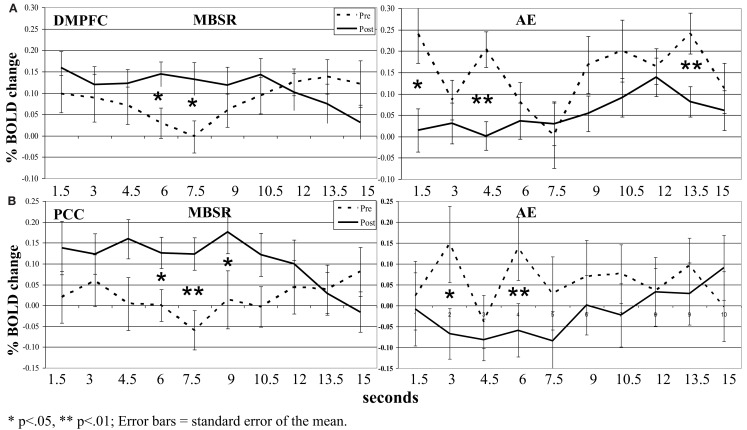
**Blood oxygen level-dependent signal time series in the (A) dorsomedial prefrontal cortex (DMPFC) and (B) posterior cingulate cortex (PCC) across each 15 s block for the contrast of negative self-view versus case pre- and post-MSBR and AE**. *p < 0.05, **p < 0.01; error bars = SEM.

In the DMPFC, there was not a three-way interaction (*p* > 0.18). However, there was a group by time interaction (*F* = 8.87, *p* < 0.005, ${\text{η}}_{\text{p}}^{\text{2}}$ = 0.18; Figure 7). Paired *t*-tests comparing BOLD response at each time point in the 15-s blocks showed significant increases from pre-to-post-MBSR in the middle of the 15-s blocks and significant decreases from pre-to-post-AE in the early and later segments of blocks.

In the VMPFC, there were no three- or two-way interactions (all *p*s > 0.35).

In the PCC, there was not a three-way interaction (*p* > 0.08). However, there was an interaction of group by time (*F* = 6.29, *p* < 0.05, ${\text{η}}_{\text{p}}^{\text{2}}$ = 0.13). Paired *t*-tests comparing BOLD response at each time point in the 15-s blocks again showed significant increases from pre-to-post-MBSR in the middle of the 15-s blocks and significant decreases from pre-to-post-AE in the early segment of blocks.

#### Treatment-related changes in whole-brain

For MBSR, a paired *t*-test yielded pre-to-post BOLD signal increases for *negative* self (versus case) in ventromedial, left ventrolateral, and bilateral dorsolateral PFC, PCC/precuneus, left inferior parietal lobule, and left posterior superior temporal gyrus (Table A1 in Appendix). The contrast of *positive* self (versus case) yielded BOLD signal increases in anterior and posterior ventromedial, dorsomedial, right ventrolateral, and left anterior dorsolateral PFC, as well as subcortical uncus. For AE, a paired *t*-test revealed no significant pre-to-post BOLD signal increases for negative self (versus case), and increased responses in only the left mid-insula for positive self (versus case Table A2 in Appendix).

## Discussion

The primary goal of this study was to investigate in the context of a RCT how MBSR, compared to an active comparison program (AE stress reduction), impacted behavioral and neural indicators of self-views in unmedicated adult patients with generalized SAD.

Clinically, both MBSR and AE significantly reduced social anxiety symptoms and disability, and enhanced mindfulness skills. Previous studies have shown similar MBSR-related improvements in patients with SAD (Goldin et al., 2009a; Goldin and Gross, 2010). While resistance and AE training have shown promise in reducing worry symptoms in patients with generalized anxiety disorders (Herring et al., 2012), this is the first study to demonstrate in an RCT that short-term (2 months) AE training reduces social anxiety symptom severity and disability inpatients with generalized SAD. The increase in mindfulness skills may be related to enhanced attention to and awareness of sensations related to instantiating a regular physical exercise practice. Some portion of the effect of AE in this study may also be related to social fear exposure occurring when exercising in a public gym facility. Ostensibly, patients with SAD may have experienced habituation or extinction of their social fears over repeated incidental exposures in the gym. Importantly, none of the self-report measures were associated with a measure of social desirability response bias, which excludes the potential confounds associated with self-report questionnaires and treatment efficacy expectations.

For the self-referential processing task (i.e., SRET), we examined self-endorsement and neural responses during positive and negative self-views. Behaviorally, MBSR and AE yielded significant self-endorsement decreases in negative and increases in positive self-views. However, compared to AE, MBSR resulted in (a) greater reduction in self-endorsement of *negative* self-views, (b) which was related to reduction in social anxiety symptom severity. Interestingly, increased mindfulness was not associated with changes in self-views. These findings suggest that chronically distorted social self-views in patients with SAD are modified during MSBR and that more adaptive self-views are related to clinical symptom improvement. Prior studies have also observed MBSR-related enhancement of more adaptive social self-views in patients with SAD (Goldin et al., 2009a).

Several mindfulness skills are trained in MBSR. One specific mindfulness skill that may modify self-views is decentering. This refers to non-identification with thoughts and beliefs, and active disengagement from ruminative self-evaluations and judgments (Fresco et al., 2007). Decentering can be understood as a cognitive process that results in attenuating habitual grasping at thoughts and beliefs as accurate reflections of the self. It has been proposed as a core mechanism of mindfulness meditation training (Segal et al., 2002; Ramel et al., 2004), that may account for the MBSR-related modification of self-views. However, the effects of decentering may manifest in different ways in different populations. In non-anxious healthy controls, decentering may appear as less identification with any self-concept. In the clinical context of SAD, however, MBSR-related increases in decentering may have a different initial impact. Specifically, in patients with SAD who have had life-long problems with inaccurate (i.e., exaggerated negative self-beliefs) and maladaptive (i.e., viewing the self as unworthy and incapable of change) self-views, MBSR might first re-establish more adaptive self-views (i.e., lesser negative, greater positive self-views) as a foundation for later inducing more subtle changes related to decentering and non-identification with any and all conceptual self-views (which is one of several more advanced result of mindfulness meditation practice).

Neurally, the manipulation check of the SRET task *at baseline* confirmed robust BOLD signal for positive and negative self-views in the expected cortical midline brain regions implicated in the SRN. Examination of differential change as a function of treatment modality revealed significant interactions during *negative* self (versus case) characterized by MBSR-related *increased* responses in the PCC and DMPFC. Examination of BOLD signal time series data showed greater recruitment of DMPFC and PCC activity in the middle portion of each 15 s block during *negative* self-processing from pre-to-post-MBSR. There was no significant effect of *positive* self (versus case). Thus, in alignment with our predictions, we did find increased attention-related PCC/precuneus response following MBSR, but did not find decreased BOLD response in more conceptual and cognitive control brain regions, as hypothesized, in the VMPFC and DMPFC.

Interestingly, recent research in developmental cognitive neuroscience suggests that SRN activation during self-reflection is considered a sign of healthy cognitive functioning and reflects the ability to sustain internal self-focused attention – an important component of self-regulation. In contrast, low levels of SRN activation have been identified as a risk factor for the development of psychological disorders and impaired social functioning (Schneider et al., 2012). Furthermore, insufficient activation of the SRN and brain networks that support socioemotional processes (specifically mentalizing, emotion, and emotion regulation) may represent disorganization and/or lack of adequate development of the self-concept (Pfeifer and Peake, 2012). Thus, activation of the SRN appears to reflect adaptive self-referential processing which may be an important component of self-development, social functioning, and self-regulation.

Further evidence for the functional relevance of the Northoff meta-analysis derived ROIs used in this study is reflected by the double dissociation of DMPFC responses (decreased during positive and increased during negative self-processing) and increased mindfulness. Increased DMPFC during negative self-processing was also related to decreases in SAD-related disability. The MBSR-related increases in DMPFC activity during negative self-processing may be related to greater ability to greater recruitment of cognitive control mechanisms that regulate habitual patterns of emotional reactivity and attention allocation. This pattern of results highlights the importance of investigating both BOLD signal magnitude and timing, as well as their relationship to individual difference measures in order to elucidate how MBSR might enhance adaptive self-focus, increase psychological flexibility and reduce clinical dysfunction.

The psychological functions instantiated in DMPFC and PCC that are recruited during the SRET following MBSR training might reveal the features of SAD that are specifically amenable to change with mindfulness meditation training. In general, increased SRN activation in this clinical context may reflect a greater engagement in the external stimulus (i.e., enhanced ability to monitor the external stimulus) without shifting to habitual automatic avoidance which is a hallmark feature of anxiety disorder. This psychological change may be a foundation for the adaptive changes in self-views as well as clinical and social functioning observed in this study.

The DMPFC has been implicated in multiple executive cognitive functions, including strategic evaluation (Lemogne et al., 2012), introspection, evaluation and decision-making (Northoff and Bermpohl, 2004; Schmitz and Johnson, 2007; van der Meer et al., 2010), cognitive discernment and explicit appraisal of self-relatedness, and cognitive reappraisal of emotional reactivity in healthy adults (Ochsner and Gross, 2005; Northoff et al., 2009). DMPFC responses have also been linked to greater ability to down-regulate negative emotions in patients with SAD (Goldin et al., 2009b). Here, increased DMPFC (along with PCC) activity during *negative* self (versus case) following MBSR may reflect increased ability to monitor present-moment experience and a concomitant reduction in prepotent cognitive biases, including attend to followed by distract away attention biases, negative interpretations, and distorted self-evaluations. Additionally, phasic increases in DMPFC may also reflect transient increases in spontaneous (i.e., uncued) cognitive reappraisal of emotional reactivity to negative self-views. Recent models suggest that enhanced MBSR-related psychological flexibility, for example, mindful coping with perceived stressors, involves the process of disengaging and withdrawing from automatic negative appraisals into a transitory metacognitive state that attenuates semantic evaluations and enhances positive cognitive reappraisal (Garland et al., 2009).

Increases in PCC/precuneus activity during *negative* self (versus case) following MBSR in adults with SAD has been reported previously (Goldin et al., 2009a). PCC/precuneus has been implicated in multiple higher-order cognitive functions, including visual-spatial imagery, episodic memory retrieval, first-person perspective taking, experience of agency, self-relevance, and personal identity (Cavanna and Trimble, 2006; Hölzel et al., 2011). Furthermore, subdivisions of the PCC/precuneus suggest different patterns of functional connectivity. In particular, the PCC and the central precuneus are known to be functionally connected with PFC regions including the DMPFC in a circuit that is related to higher-order executive processing (Margulies et al., 2009). A recent review suggests that the MPFC and PCC are part of a brain network with other brain regions implicated in mentalizing (i.e., theory of mind), emotion awareness and emotion regulation, and that the functioning of these psychological functions are fundamental in the development of socioemotional skills and self-concept (Pfeifer and Peake, 2012). Clinically, concomitant increases in DMPFC and PCC have been linked to successful pharmacologic treatment (Samson et al., 2011) and interpersonal therapy for major depression (Martin et al., 2001). This is the first demonstration of increased DMPFC and PCC being linked to clinical improvement in patients with SAD following MBSR.

The whole-brain analysis was not the primary focus of this study. However, the whole-brain results provided further support that MBSR training increased activity in brain regions involved in cognitive regulation (medial PFC, bilateral dorsolateral PFC, left ventrolateral PFC), and attention regulation (right DLPFC, PCC/precuneus, inferior parietal lobule; Fan et al., 2005). Here, even though there was no explicit instruction to implement top-down regulation during the SRET task, these brain changes suggest that MBSR enhanced the recruitment of cognitive and attention regulation brain networks that reduce habitual automatic emotional (i.e., anxiety and fear) and behavioral (e.g., avoidance, attention disengagement) patterns. This converges with proposed mechanisms of MBSR, namely, enhanced attention regulation and cognitive regulation of emotion, as well as modulation of self-views (Hölzel et al., 2011). It further suggests that increases in cognitive regulation-related PFC regions (as was seen in the Farb et al., 2007 study during experiential versus narrative self-focus), might facilitate modification of habitual self-views.

With regard to the effect of AE, there was clear evidence of enhanced positive and reduced negative self-views, albeit less than MBSR, as well as similar AE and MBSR improvement in clinical symptoms and mindfulness skills. What is less clear, however, is what psychological and neural mechanisms are modulated by AE that account for these changes. Given the completely different pattern of pre-to-post-AE and MBSR SRN ROIs (Figures 6 and 7) and whole-brain (Tables A1 and A2 in Appendix) changes, different brain mechanisms must be at play. Clearly, MBSR-related processes such as decentering or non-identification with thought and beliefs are not likely to be the skills actively cultivated with AE. The increased left insula activity for positive self versus case could suggest greater viscerosomatic awareness following AE. From our previous published report (Jazaieri et al., 2012), we know that AE (and MBSR) were associated with significant increases in self-esteem, and that increased self-esteem is associated with pre-to-post-AE decreases in social anxiety symptoms (*r* = 0.58, *p* < 0.05) increases in use of cognitive reappraisal (*r* = 0.57, *p* < 0.05) and increases in cognitive reappraisal self-efficacy (*r* = 0.68, *p* < 0.01). As noted above, repeated social fear exposure during regular visits to the gym facility may also be related to the changes in self-esteem and associations with social anxiety symptoms, cognitive reappraisal, and self-views.

The most important implications of this study are that MBSR can modulate self-views in ways that are clearly detectable via self-report and brain networks, especially for negative self-views, and that the self-report and neural changes are related to important indicators of social anxiety symptom severity and disability. Furthermore, this study highlights that MBSR-related increases in the SRN may be an important first step in re-establishing adaptive self-views and social functioning in patients with SAD.

The present study compared MBSR to AE, another active control stress reduction training intervention. One disparity is that while MBSR involves learning new mindfulness skills, AE does not. Also, in future studies, adding a no training waitlist control group could be useful to examine habituation to the SRET task. Nonetheless, the significant group by time interactions of self-endorsement ratings and BOLD responses only for negative self-views indicate meaningful differential changes from pre-to-post-MBSR versus AE. The SRET task implemented here used experimenter-selected positive and negative social trait adjectives selected for their relevance to SAD. Use of participant-generated (i.e., idiographic) stimuli may result in more robust brain-behavioral responses in patients with SAD and perhaps larger differential effects of MBSR versus AE. The duration of each word stimulus was only 3 s. Use of longer stimulus durations will allow for examination of differences in self-focused attention, and rumination, as well as temporal features of BOLD responses in the SRN. This study only used single adjectives as stimuli. Future studies could explore adding non-verbal cues associated with positive and negative (and perhaps neutral) self-views. The present study used binary yes/no responses for the target condition (self-descriptive) and control condition (upper case letters). Future studies will benefit from using a continuous rating scale to obtain more refined measurement of how self-descriptive a target social trait is perceived to be.

## Conflict of Interest Statement

The authors declare that the research was conducted in the absence of any commercial or financial relationships that could be construed as a potential conflict of interest.

## Appendix

**Table A1:** **Pre-to-post-mindfulness-based stress reduction changes in BOLD responses for negative and positive self versus case**.

Brain regions	BA	*xyz*	%	Vol mm^3^	*t*-Test
**MBSR post versus****pre**					
**Negative self > case**					
**FRONTAL LOBES**					
*Rostromedial PFC*	10	−3 62 8	0.16	957	3.56
*Caudomedial PFC*	10	7 52 −2	0.12	426	3.21
L middle frontal gyrus	8	−38 35 50	0.22	904	3.15
R middle frontal gyrus	8	31 31 46	0.12	1223	3.29
L Ventrolateral PFC	47	−38 31 −2	0.14	1011	3.47
**TEMPORAL LOBES**					
L superior temporal gyrus	22	−69 −13 1	0.15	213	4.18
**PARIETAL LOBES**					
*PCC/precuneus*	31/7	0 −61 29	0.10	1277	3.62
L inferior parietal lobule	40	−45 −61 46	0.21	426	3.66
**Negative case > self**	None				
**Positive self > case**					
**FRONTAL LOBES**					
*Rostromedial PFC*	10	0 62 12	0.18	851	4.64
*Dorsomedial PFC*	8	3 52 50	0.41	372	3.46
L rostral superior frontal gyrus	9	−17 52 29	0.08	319	3.65
Caudal rectal gyrus	25	7 4 −16	0.24	319	3.55
R ventrolateral PFC	46	58 31 12	0.14	213	3.36
**SUBCORTICAL**					
L uncus	28	−31 4 −19	0.08	319	3.15
**Positive case > self**	None				

**Italicized* brain regions are located within the Northoff defined self-referential cortical midline network. *xyz* = Talairach coordinates for peak of cluster, % = BOLD percent signal change for contrast, *t*-value threshold ≥3.10, voxel *p* < 0.005, minimum cluster volume threshold >213 mm^3^ (4 voxels × 3.438 mm × 4.5 mm), cluster-wise *p* < 0.01. BA, Brodmann area; BOLD, blood oxygen level-dependent; L, left; MFG, middle frontal gyrus; PCC, posterior cingulate cortex; PFC, prefrontal cortex; R, right; SAD, social anxiety disorder; Vol, volume*.

**Table A2:** **Pre-to-post aerobic exercise changes in BOLD responses for negative and positive self versus case**.

Brain regions	BA	*xyz*	%	Vol mm^3^	*t*-Test
**AE post versus****pre**					
**Negative self > case**	None				
**Negative case > self**					
**FRONTAL LOBES**					
*Dorsal medial PFC*	8	0 25 43	−0.17	479	−3.64
*Medial PFC*	9	0 49 15	−0.14	213	−3.34
*Rostral anterior cingulate*	24	3 27 1	−0.17	479	−3.59
L ventral lateral PFC	47	−31 25 −2	−0.10	319	−4.66
L ventral lateral PFC	47	−48 31 −9	−0.27	213	−3.27
R inferior frontal gyrus	45	41 21 12	−0.10	213	−4.17
**PARIETAL LOBES**					
R lateral precuneus	7	28 −41 43	−0.14	266	−3.39
**OCCIPITAL LOBES**					
R lingual gyrus	18	3 −79 −9	−0.20	798	−4.68
L lingual gyrus	18	−7 −65 5	−0.11	532	−3.28
L lingual gyrus	18	−3 −79 −6	−0.19	213	−4.16
**SUBCORTICAL**					
R caudate		3 1 12	−0.17	745	−3.40
R caudate		14 11 5	−0.12	479	−4.84
R caudate		17 −10 22	−0.09	213	−3.71
R thalamus		14 −10 1	−0.10	426	−4.76
**Negative case > self**	None				
**Positive self > case**					
L insula	13	−31 −10 15	0.07	213	3.65
**Positive case > self**	None				

**Italicized* brain regions are located within the Northoff defined self-referential cortical midline network. *xyz* = Talairach coordinates for peak of cluster, % = BOLD percent signal change for contrast, *t*-value threshold ≥3.10, voxel *p* < 0.005, minimum cluster volume threshold >213 mm^3^ (4 voxels × 3.438 mm × 4.5 mm), cluster-wise *p* < 0.01. BA, Brodmann area; BOLD, blood oxygen level-dependent; L, left; PFC, prefrontal cortex; R, right; SAD, social anxiety disorder; Vol, volume*.
